# Addressing the Antibiotic Resistance Problem with Probiotics: Reducing the Risk of Its Double-Edged Sword Effect

**DOI:** 10.3389/fmicb.2016.01983

**Published:** 2016-12-15

**Authors:** Ivan C. V. J. Imperial, Joyce A. Ibana

**Affiliations:** Immunopharmacology Research Laboratory, Institute of Biology, College of Science, University of the Philippines DilimanQuezon City, Philippines

**Keywords:** antibiotic resistance, probiotics, mobile genetic elements, veterinary medicine, livestock production

## Abstract

Antibiotic resistance is a global public health problem that requires our attention. Indiscriminate antibiotic use is a major contributor in the introduction of selective pressures in our natural environments that have significantly contributed in the rapid emergence of antibiotic-resistant microbial strains. The use of probiotics in lieu of antibiotic therapy to address certain health conditions in both animals and humans may alleviate these antibiotic-mediated selective pressures. Probiotic use is defined as the actual application of live beneficial microbes to obtain a desired outcome by preventing diseased state or improving general health. Multiple studies have confirmed the beneficial effects of probiotic use in the health of both livestock and humans. As such, probiotics consumption is gaining popularity worldwide. However, concerns have been raised in the use of some probiotics strains that carry antibiotic resistance genes themselves, as they have the potential to pass the antibiotic resistance genes to pathogenic bacteria through horizontal gene transfer. Therefore, with the current public health concern on antibiotic resistance globally, in this review, we underscore the need to screen probiotic strains that are used in both livestock and human applications to assure their safety and mitigate their potential in significantly contributing to the spread of antibiotic resistance genes in our natural environments.

## Introduction

Since its advent, antibiotics remain as the major therapeutic strategy that is used to address numerous diseases of infectious etiologies both in human and veterinary medicine (Drago et al., 2011; Schjørring and Krogfelt, 2011; Verraes et al., 2013; Allen and Stanton, 2014; Card et al., 2015). However, the indiscriminate and improper use of antibiotics has led to the decreased susceptibility and increased resistance rates observed not only in disease-causing microbes but in commensal microbes as well (Rosander et al., 2008; Drago et al., 2011; Allen and Stanton, 2014; von Wintersdorff et al., 2014; Card et al., 2015). Rampant antibiotic use has pushed microbes to adapt and survive by acquiring antibiotic resistance genes that led to antibiotic-resistant strains (Schjørring and Krogfelt, 2011; Forslund et al., 2013; Fouhy et al., 2013; Ghosh et al., 2013; Verraes et al., 2013; Allen and Stanton, 2014; Card et al., 2014). Antibiotic resistance genes are then vertically passed on to the next generation of microbes; in some cases, they are acquired through horizontal transfer from one microbe to another, when thriving in the same microbial environment (Schjørring and Krogfelt, 2011; Broaders et al., 2013; Forslund et al., 2013; Fouhy et al., 2013; Ghosh et al., 2013; Verraes et al., 2013; Allen and Stanton, 2014; Card et al., 2014, 2015).

In the human clinical setting, these antibiotic-resistant pathogens have caused numerous treatment failures that eventually led to both hospital morbidities and mortalities (Vankerckhoven et al., 2008; Schjørring and Krogfelt, 2011; Lu et al., 2014). Overall, the prevalence of antibiotic resistance has now become a global health problem that needs urgent attention from the world health authorities (Egervärn et al., 2010; Schjørring and Krogfelt, 2011; Ghosh et al., 2013; Penders et al., 2013; Hu et al., 2014; Lu et al., 2014; von Wintersdorff et al., 2014; Card et al., 2015; van Schaik, 2015). In addressing the problem on antibiotic resistance, the use of probiotics in lieu of antibiotics for treating certain diseases of host organisms has been investigated (Rosander et al., 2008; Muñoz-Atienza et al., 2013). Numerous studies have shown that instead of killing pathogenic microbes through antibiotics, the establishment of commensal and sometimes mutualistic microbes may hinder the growth of disease-causing microbes found in the same host microbial environment (Saarela et al., 2007; Hammad and Shimamoto, 2010; Klein, 2011; Nueno-Palop and Narbad, 2011; Wei et al., 2012; Varankovich et al., 2015). In addition, it has also been demonstrated that maintaining what is considered “normal” microbiota for certain host microbial environments may prevent diseased conditions that are not necessarily of infectious etiology and may improve general health outcome (Franz et al., 2011; Nueno-Palop and Narbad, 2011; Wei et al., 2012; Téllez et al., 2015; Varankovich et al., 2015). As a result, probiotic use, defined as the application of actual live beneficial microbes, has been increasingly practiced for both human and veterinary applications (Tompkins et al., 2008; Vankerckhoven et al., 2008; Sanders et al., 2010; Xiao et al., 2010; Nueno-Palop and Narbad, 2011; Songisepp et al., 2012; Devi et al., 2015; D’Orazio et al., 2015; Fuochi et al., 2015; Senan et al., 2015; Varankovich et al., 2015). Among the modes of probiotic use, the consumption of probiotics through the gastrointestinal route may be considered the most common application in both human and veterinary uses.

However, microbes used as probiotics are not exempted from acquiring antibiotic resistance genes. Given their shared microbial environment in the gastrointestinal tract, a risk of pathogenic microbes acquiring antibiotic resistance genes from probiotic microbes exists, and vice versa (Mater et al., 2007; Rosander et al., 2008; Liu et al., 2009; Egervärn et al., 2010; Drago et al., 2011; Nueno-Palop and Narbad, 2011; Gueimonde et al., 2013; Varankovich et al., 2015). If improperly cooked, livestock treated with probiotics that are consumed by humans as food may also pose as a possible source of antibiotic resistance genes for the human gut microbiota (Devirgiliis et al., 2011; Schjørring and Krogfelt, 2011; Forslund et al., 2013; Verraes et al., 2013; Allen and Stanton, 2014; Hu et al., 2014; Woolhouse et al., 2015). To complicate the aforementioned risks, some probiotic microbes are even screened specifically for antibiotic resistance to be used concomitantly with antibiotics in treating certain medical conditions (Galopin et al., 2009; Hammad and Shimamoto, 2010). As such, there is a need to review existing studies to clarify the safety of increasing probiotic use in relation to the existence of antibiotic resistance genes.

This review aims to describe the processes that govern the spread of antibiotic resistance in relation to antibiotic resistance genes. Antibiotic resistance gene transfer in the absence of probiotics is discussed first to elucidate the ongoing problem of the prevalence of antibiotic-resistant bacterial strains. Probiotic uses in both human and veterinary applications are then described and reviewed to reaffirm their beneficial use. Screening of probiotic bacterial strains for antibiotic resistance genes is then discussed to evaluate the safety of probiotic use. Finally, probiotic use in relation to the spread of antibiotic resistance genes is tackled to clarify the potential role of probiotics in propagating antibiotic resistance.

## Antibiotic Resistance

Although the remarkable increase in the incidence and prevalence of antibiotic resistance were observed after the introduction and widespread use of antibiotics (Datta and Hughes, 1983; Hughes and Datta, 1983), antibiotic resistance is believed to have existed long before human antibiotic use (Hughes and Datta, 1983; Broaders et al., 2013). It is evident in multiple ecological interactions, wherein many organisms, may they be microbes or macro-organisms, have the ability to produce natural antibiotics that ultimately increase their chances of survival (Samuels et al., 2013; Cawoy et al., 2014; Timbermont et al., 2014; Pinchas et al., 2015; Sherpa et al., 2015; Téllez et al., 2015). Organisms use antibiotics to kill or inhibit growth of pathogenic microbes, while some microbes use antibiotics to compete for the same resources that other microbes consume as well (Samuels et al., 2013; Timbermont et al., 2014; Pinchas et al., 2015; Sherpa et al., 2015; Téllez et al., 2015). As a natural evolutionary response, microbes that are able to adapt to and survive these natural antibiotics gain advantage in producing the next generation of microbes. Hence, antibiotic resistance is a natural phenomenon (Sherpa et al., 2015).

Taking advantage of these ecological interactions, we have learned to harness the use of antibiotics for a variety of applications (Scanlon et al., 2014; Sherpa et al., 2015). We have used antibiotics to treat infectious diseases that were once considered very fatal, until the advent of readily available antibiotic medications (Sherpa et al., 2015). In agriculture, we have learned that the use of antibiotics greatly increases yield in rearing animals primarily as food source (Allen and Stanton, 2014; Woolhouse et al., 2015; Xiao et al., 2015). Although farmers were not entirely clear as to how antibiotic use increases growth rates of livestock animals, they have continually used antibiotics in sub-therapeutic doses as feeding supplements with observable results (Allen and Stanton, 2014). In addition, infectious diseases that once plagued farm animals are now treated successfully by antibiotics and even prevented by prophylactic use (Allen and Stanton, 2014; Woolhouse et al., 2015). As such, humans have multiple reasons to use antibiotics.

However, with rampant antibiotic use, the natural way of developing antibiotic resistance has also changed in both manner and rate. Due to different human applications, multiple avenues for microbes to encounter antibiotics have put immense selective pressure on microbes to develop antibiotic resistance (Rosander et al., 2008; Devirgiliis et al., 2011; Drago et al., 2011; Schjørring and Krogfelt, 2011; Forslund et al., 2013; Fouhy et al., 2013; Ghosh et al., 2013; Verraes et al., 2013; Allen and Stanton, 2014; Hu et al., 2014; von Wintersdorff et al., 2014; Card et al., 2015). In turn, these have hastened the development of antibiotic-resistant microbial strains. The rate of developing antibiotic-resistant strains is now occurring much faster than the rate of discovering new antibiotics (Sherpa et al., 2015).

### Antibiotic Resistance Genes and the Influence of Ecology

Just like any evolutionary response, adaptive changes of an organism are passed on to the next generation via the organism’s genetic material. Some microbes possess antibiotic resistance genes that confer their ability to survive exposure to antibiotics (Rosander et al., 2008; Drago et al., 2011; Schjørring and Krogfelt, 2011; Forslund et al., 2013; Fouhy et al., 2013; Ghosh et al., 2013; Verraes et al., 2013; Allen and Stanton, 2014; Hu et al., 2014; von Wintersdorff et al., 2014; Card et al., 2015). According to the Antibiotic Resistance Gene Database, there are at least 23,317 antibiotic genes established so far, which are effective against at least 249 known antibiotics (Hu et al., 2013). In several recent studies, investigators have shown that the presence of antibiotic resistance genes in microbial organisms is greatly affected by their microbial ecology (Forslund et al., 2013; Hu et al., 2013, 2014; Gibson et al., 2015). This observation reiterates the natural selection pressure toward antibiotic resistance brought about by ecological interactions found in the natural microbial environment (Sherpa et al., 2015).

In the study of Hu et al. (2013), investigators studied the presence of antibiotic resistance genes in the human gut microbiota. A total of 162 individuals from China, Denmark, and Spain were screened for antibiotic resistance genes. It was found that a total of 1,093 antibiotic resistance genes were present in the sample population. Antibiotic resistance genes against three antibiotic classes – tetracycline, macrolides, and beta-lactams – accounted for more than 75% of the total antibiotic resistance genes. Interestingly, antibiotic resistance genes found in Chinese individuals were statistically clustered in terms of similarity, when compared to the two European populations that had statistically more similar antibiotic resistance profiles. This suggests that the actions of human hosts as a population also affect the microbial ecology and, ultimately, the antibiotic resistance gene profiles of the gut microbiota. In support of the previous statement, a study of Lu et al. (2014) demonstrated that human populations of different age groups also produced different antibiotic resistance genes profiles that were statistically clustered by age. Hence, different activities of different human age groups also dictate antibiotic resistance profiles of human gut microbiota.

In addition, Forslund et al. (2013) showed that country-specific antibiotic use influences the antibiotic resistance genes found in the human gut microbiota. In addition to actual medical application of antibiotics, the investigators also considered the antibiotics used in livestock agriculture for food production. Investigators found that veterinary use of antibiotics also influenced antibiotic resistance genes profile of the human gut microbiota. This reaffirms the role of ecological interactions among humans, animals, and the microbial environment in influencing antibiotic resistance genes, ultimately found in the human gut microbiota (Schjørring and Krogfelt, 2011; Hu et al., 2014). As such, many studies recommend that in addressing the problem on antibiotic resistance, an ecological approach and perspective are needed as evidence suggests that antibiotic resistance is not entirely confined to human medical antibiotic use alone (Devirgiliis et al., 2011; Schjørring and Krogfelt, 2011; Forslund et al., 2013; Verraes et al., 2013; Allen and Stanton, 2014; Hu et al., 2014; Woolhouse et al., 2015). Agricultural use of antibiotics should be properly regulated as well as clinical prescription of antibiotics in humans (Verraes et al., 2013; Allen and Stanton, 2014; Woolhouse et al., 2015).

### Antibiotic Resistance Genes and Their Spread through Mobile Genetic Elements

As mentioned earlier, antibiotic resistance genes are vertically transferred from one generation to another and favor the survival of resistant microbes (Schjørring and Krogfelt, 2011; Penders et al., 2013; Verraes et al., 2013; Allen and Stanton, 2014; Fouhy et al., 2014; Hu et al., 2014; Card et al., 2015; van Schaik, 2015). However, it is the ability of microbes to conduct horizontal gene transfer that somehow complements the rampant human antibiotic applications in hastening the development of antibiotic-resistant strains. Horizontal gene transfer in microbes is made possible through mobile genetic elements (Karami et al., 2007; Mater et al., 2007; De Vries et al., 2011; Drago et al., 2011; Haug et al., 2011; Broaders et al., 2013; Machado and Sommer, 2014; von Wintersdorff et al., 2014). The gastrointestinal tract is a prime candidate for conducting studies on horizontal gene transfer (Broaders et al., 2013).

In the human gastrointestinal tract, four mobile genetic elements are considered: plasmids, conjugative transposons, integrons, and bacteriophages (Broaders et al., 2013). Among mobile genetic elements, only bacterial plasmids, conjugative transposons, and integrons are considered well-established and well-documented to cause antibiotic genes transfer in both the natural environment and the clinical setting. It has been reported that antibiotic resistance genes were successfully transferred from a commensal bacterial strain to a pathogenic bacterial strain, and vice versa, using plasmids as vectors of horizontal gene transfer (Broaders et al., 2013). Using conjugative transposons, antibiotic resistance to tetracycline, chloramphenicol, kanamycin, and erythromycin are reported to be transferred in certain species of bacteria. The loss of efficacy of tetracycline against opportunistic *Bacteroides* spp. infection is specifically attributed to conjugative transposons (Broaders et al., 2013). Integrons, which function very similarly, like transposons, have been identified as the cause of spread of antibiotic resistance in *Vibrio cholerae* (Broaders et al., 2013). On the other hand, the role of bacteriophages in promoting bacterial horizontal gene transfer is only suggested with the small but significant presence of phage-related proteins in the human gut microbial community (Broaders et al., 2013). Given the genetic-hacking machinery of viruses, bacterial horizontal gene transfer brought about by bacteriophage infection from one bacterium to another cannot be simply ruled out.

Besides mobile genetic elements, it was suggested that perhaps the most mobile that antibiotic resistance genes can be is through actual travel of human hosts carrying antibiotic-resistant strains. In the study of von Wintersdorff et al. (2014), the investigators demonstrated that international travel of 122 healthy Dutch travelers with documented fecal antibiotic resistance genes profiles caused significant increases in extended spectrum beta-lactamase-encoding genes and quinolone resistance-encoding genes found in their gut microbiota, immediately after their return to the Netherlands. Travel to Southeast Asia and travel to the Indian subcontinent were the most associated with increase in quinolone resistance-encoding genes alone, and increase in both beta-lactamase-encoding genes and quinolone resistance-encoding genes, respectively. In sum, these alarming findings suggest that human travel can contribute to the global spread of antibiotic resistance genes (von Wintersdorff et al., 2014).

### Current Efforts to Control Antibiotic Resistance

Given the ecological nature of the problem on antibiotic resistance, several nations have adopted policies to address the issue. Perhaps the most notable is that of the member states of the European Union (EU). Since 2006, all EU countries have prohibited the use of antibiotics for the sole purpose of growth promotion in agricultural livestock industry (Cogliani et al., 2011). In addition, the European Food Safety Authority (EFSA) has instituted guidelines on the use of food additives in animal products that may potentially spread antibiotic resistance genes (Panel, 2012). However, other developed nations, such as the United States (US), has not imposed strict regulatory policies on antibiotic use for livestock growth promotion. Animal food and pharmaceutical industries in the US have strongly opposed restrictions in antibiotic use and have argued that such policies have been detrimental to food production in places where they were implemented. Furthermore, some countries do not have any known or established policy regarding the use of antibiotics as growth promoters, nor even require veterinary prescription for animal antibiotic use (Maron et al., 2013).

With the current paucity of clear regulatory policies in many countries, a global effort is needed in order to consolidate an effective strategy in controlling antibiotic resistance. Without a unifying regulatory guideline to follow, the problem on antibiotic resistance will most likely persist given the interweaving ecological interactions that govern the spread of antibiotic resistance. Furthermore, the spread of antibiotic resistance genes through human travel or trading of animal products between nations may make instituted regulatory policies of some countries less effective.

## Use of Probiotics

As the development of antibiotic resistance strains continues, the use of probiotics as a substitute for antibiotics is becoming more popular in both the medical field and livestock agriculture (Collins and Gibson, 1999; Collado et al., 2012; Muñoz-Atienza et al., 2013; D’Orazio et al., 2015; Téllez et al., 2015; Varankovich et al., 2015). Probiotic use is defined as the actual application of live beneficial microbes to obtain a desired outcome, may it be prevention of a diseased state or improvement in general health outcome observed in the host organism (Collins and Gibson, 1999). The basis of its efficacy relies on the symbiosis of an established microbial ecology that resists the intrusion or overproduction of pathogens that lead to a diseased condition of the host organism (Catanzaro and Green, 1997). By limiting the use of antibiotics, probiotic use may help decrease the rate of development of antibiotic-resistant strains secondary to widespread and rampant antibiotic use (Collins and Gibson, 1999; Collado et al., 2012; Muñoz-Atienza et al., 2013; Varankovich et al., 2015).

### Probiotics and Livestock Benefits

Many farm animals are considered to be adapted in a symbiotic relationship with microbes as shown by specialized gastrointestinal organs that enable microbial fermentation (Téllez et al., 2015). The capacity of farm animals to ferment complex polysaccharides with the help of their intestinal microbiota gives as much as 70% of energy acquired by ruminants and up to 30% of energy acquired by monogastric animals (Téllez et al., 2015). However, it is only fairly recent that this symbiotic relationship between farm animals and their microbiota is taken advantage of by farmers with the use of probiotics. With the increasing problem on antibiotic resistance, studies have shown that probiotic use can replace antibiotics in preventing diseased conditions and promoting growth in livestock animals (Muñoz-Atienza et al., 2013; Téllez et al., 2015).

Probiotic use in livestock agriculture of chickens and turkeys has been demonstrated to confer increased resistance in *Salmonella* spp. infections through accelerated establishment of what is considered normal and healthy microbiota for the aforementioned birds (Téllez et al., 2015). In addition, the incidence of idiopathic diarrhea in commercial turkey brooding houses was reported to be decreased by probiotic use (Téllez et al., 2015). As a whole, large-scale commercial trials of appropriate probiotic administration in chickens and turkeys demonstrated increased performance and reduced overall costs of production (Téllez et al., 2015).

In cattle raising, it was reported that probiotic use provided no significant effects in reducing cattle pathogens (Téllez et al., 2015). However, *Escherichia coli* O157:H7, a food-borne pathogen capable of causing severe hemorrhagic illness in humans, was known to be shed by livestock animals in their feces. With the use of different combinations of bacteria used as probiotics, it was shown that there was decreased shedding of *E. coli* O157:H7 in both cattle and sheep livestock that may translate to decreased risk of *E. coli* O157:H7 human infections (Téllez et al., 2015).

Even in a different habitat such as in aquaculture, probiotic use has also been gaining ground. In the study of Muñoz-Atienza et al. (2013), it was demonstrated that lactic acid-producing bacteria of aquatic origin can exhibit antimicrobial activity against established gram-positive and gram-negative fish pathogens. However, no comprehensive *in vivo* assessment has yet to determine the beneficial and possible harmful effects of probiotic use in aquaculture (Muñoz-Atienza et al., 2013).

### Probiotics and Human Health

For humans, probiotic use has become a popular practice for promoting good health. Many commercial food products are now being supplemented by probiotic bacteria with claims of promoting good health (Songisepp et al., 2012). It is no surprise that there is an increasing number of probiotic users as we begin to understand better the role and importance of human gut microbiota, as well as what is considered to be “normal” and symbiotic human gut microbiota (Catanzaro and Green, 1997; Collins and Gibson, 1999; Collado et al., 2012).

The complex microbiota of the human gut is considered to play important roles in several gastrointestinal functions. These functions include host nutrition, regulation of gut epithelial development, regulation of fat storage, stimulation of intestinal angiogenesis, inflammatory immune response, and pathogen resistance (Collins and Gibson, 1999; Ventura et al., 2009). In parallel comparisons with the macro-ecosystems of the natural environment, we can deduce that the more stable the composition of the human gut microbiota is, the more beneficial it is for the human host habitat. Indeed, there are expected human gut microbiota profiles that are considered to be symbiotic and are indicators of good health (Varankovich et al., 2015). This is supported by associations demonstrated between deviations in the composition of symbiotic adult pattern of human gut microbiota and a variety of human medical conditions such as inflammatory bowel disease, allergy, obesity, and atopic disease (Ventura et al., 2009).

Looking at current human medical applications, probiotic use is clinically proven to modulate infant gut microflora disturbance after antibiotic use (Collado et al., 2012). The use of antibiotics early in the infant’s life is considered detrimental to infant gut microbial diversity, as antibiotics do not discriminate in killing microorganisms that are affected by the antibiotics’ mechanism of action. As such, normal gut microbiota composed of commensal and beneficial microbes is decreased in the infant’s gut, allowing the potential increase in the population of harmful microbes that are not affected by the antibiotics’ mechanism of action. This process leads to diseased conditions associated with antibiotic use such as antibiotic-induced diarrhea (Collins and Gibson, 1999; Collado et al., 2012; Varankovich et al., 2015). In addition, perinatal and postnatal probiotic use are reported to have potential benefits in preventing future developments of allergies (Kukkonen et al., 2007), asthma (Luoto et al., 2010), gastrointestinal infections (Johnston et al., 2007), and obesity (Vliagoftis et al., 2008), as the aforementioned diseased conditions are all associated with the interplay of gut microbiota and the development of the host immune system (Collado et al., 2012). By disturbing the infant’s gut microbiota, antibiotics pose a potential risk in pre-disposing infants to the aforementioned diseased conditions.

Unlike in infants, human adults with consistent oral intake of established probiotics have not yet shown clinically significant changes in adult gut microbiota composition, structure, and gene content (Ursell et al., 2013). However, for a specific medical condition, such as *Clostridium difficile*-associated diarrhea, studies have shown that probiotic use, sometimes in the extreme form of fecal transplantation, effectively addresses recurrent *C. difficile*-associated diarrhea (Ursell et al., 2013; Van den Abbeele et al., 2013; Varankovich et al., 2015). *C. difficile*-associated diarrhea is a disease associated with prolonged antibiotic use which kills normal human gut microbiota that supposedly hinders the growth of the *C. difficile* population (Van den Abbeele et al., 2013; Varankovich et al., 2015).

There are other human medical conditions now being connected to the human gut microbiota. Interestingly, these medical conditions associated with human gut microbiota are not confined to diseases with infectious etiologies (Ventura et al., 2009). Common medical conditions with established non-microbial pathophysiologies, such as obesity and diabetes mellitus, and much rarer gastrointestinal disorders, such as irritable bowel syndrome, Crohn’s disease, and necrotizing enterocolitis, are presently being associated with dysbiosis in the human gut microbiota (Peterson et al., 2008; Emami et al., 2009; Serino et al., 2009; Salonen et al., 2010; Angelakis et al., 2012; Snedeker and Hay, 2012; Jeffery and O’Toole, 2013). Deviation from what is accepted to be “normal” human gut microbial ecology appears to be part of the aforementioned diseases’ pathophysiologies. Therefore, more medical applications of probiotic use to maintain normal human gut microbial ecology are anticipated to appear in the near future.

## Safety of Probiotic Use

Microbes used as probiotics are not exempted from the natural processes governing antibiotic resistance (Mater et al., 2007; Rosander et al., 2008; Chang et al., 2009; Egervärn et al., 2010; Drago et al., 2011; Nueno-Palop and Narbad, 2011; Gueimonde et al., 2013; Varankovich et al., 2015).

As such, it is imperative to screen microbes effectively for antibiotic resistance genes before using them as probiotics. A crucial aspect in studying antibiotic resistance in probiotic bacteria is to separate intrinsic resistance from acquired resistance (Sanders et al., 2010). Mechanisms of intrinsic resistance, such as active eﬄux of antibiotics by a bacterial outer membrane, is not governed by acquired antibiotic resistance genes (Chang et al., 2009; Hammad and Shimamoto, 2010). Focusing on acquired antibiotic resistance, random genetic changes on chromosomal genes should be further distinguished from the more likely transmissible type of antibiotic resistance.

However, to our knowledge, no unified world-wide health authority has taken full responsibility in screening for antibiotic resistance genes in probiotic microorganisms. Fortunately, research projects, such as the Biosafety Assessment of Probiotics used for Human Consumption (PROSAFE), the Assessment and Critical Evaluation of Antibiotic Resistance Transferability in Food Chain (ACE-ART), and the Joint International Organization for Standardization-International Dairy Federation Action Team on Probiotics (ISO-IDF) have individually contributed to address the issue (Sanders et al., 2010). In evaluating the safety of potential probiotic strains, two statuses are currently acceptable – Qualified Presumption of Safety (QPS) by the EFSA and Generally Recognized as Safe (GRAS) by the US-FDA. It is noteworthy that GRAS status is applied to microorganisms and microbial-derived ingredients used in food products while QPS is applied to any biological agent in the form of bacteria, fungi, or virus, that is intentionally added at different stages into the food chain. However, the QPS is considered by many as the more applicable and flexible criteria, given the emerging risk of spreading antibiotic resistance genes through probiotic strains (Sanders et al., 2010).

In the paper of Sanders et al. (2010), a case was discussed wherein the outcomes of aforementioned standards for evaluating potential probiotics were not consistent. The case involved the use of a probiotic bacteria intended for infant-formula that claims improved growth in developing infants. Both standards were aware that the probiotic strain involved carries a chromosomal gene for tetracycline resistance. Using the GRAS criteria, the US FDA evaluated the “safety of probiotic use” with “reasonable certainty,” while the European counterpart cited “safety of probiotic use in light of the strength or weakness of the evidence for benefit” and “the lack of knowledge necessitates application of precautionary principles.” As such, the US FDA granted GRAS status to the involved probiotic strain, while the EFSA did not grant QPS status.

Given the separate approaches in screening probiotic bacterial strains, multiple independent studies have demonstrated several methods in screening different probiotic strains for antibiotic resistance genes (Tompkins et al., 2008; Vankerckhoven et al., 2008; Chang et al., 2009; Galopin et al., 2009; Xiao et al., 2010; Klein, 2011; Nueno-Palop and Narbad, 2011; Cebrián et al., 2012; Songisepp et al., 2012; Wei et al., 2012; Tan et al., 2013; Devi et al., 2015; Senan et al., 2015). In places where it is not bound to either US FDA or EFSA, screening for antibiotic resistance genes in probiotic strains becomes even more important as rigid guidelines and regulations for probiotic use are lacking (Chang et al., 2009).

### Lactic Acid Bacteria and Their Safety Profile

The most common group of bacteria used as probiotics belongs to the group of lactic acid bacteria (Gueimonde et al., 2013; Varankovich et al., 2015). Included in this group are *Lactobacillus* and *Enterococcus.* Both genera are currently extensively screened for species that can be used as probiotic bacteria (Tompkins et al., 2008; Vankerckhoven et al., 2008; Chang et al., 2009; Klein, 2011; Nueno-Palop and Narbad, 2011; Cebrián et al., 2012; Songisepp et al., 2012; Tan et al., 2013; Devi et al., 2015; Senan et al., 2015).

*Lactobacillus* probiotic strains are capable of improving digestion, absorption, and availability of nutrients in both livestock animals and humans (Téllez et al., 2015; Varankovich et al., 2015). They are also known to inhibit and kill *Helicobacter pylori*, a pathogen regarded as the major cause of gastritis and peptic ulcers, and is a risk factor for gastric malignancy in humans (Varankovich et al., 2015). In addition, the risk of human infectious disease due to *Lactobacillus* is considered negligible at less than one case per million individuals (Sanders et al., 2010).

*Enterococcus* probiotic strains are also known to be effective in reducing recovery periods of acute diarrhea in both animals and humans (Vankerckhoven et al., 2008; Varankovich et al., 2015). However, unlike *Lactobacillus*, the genus *Enterococcus* has member strains that are considered opportunistic pathogens and are sometimes the etiologic agents of some human nosocomial infections, such as bacteremia and infective endocarditis (Vankerckhoven et al., 2008; Sanders et al., 2010; Franz et al., 2011; Varankovich et al., 2015).

It has also been reported in several studies that some species of lactic acid bacteria have intrinsic resistance to bacitracin, kanamycin, teicoplanin, vancomycin, and beta-lactams (Varankovich et al., 2015). Given the beneficial effects of lactic acid bacteria, intrinsic resistance to certain antibiotics may be considered advantageous if an antibiotic-probiotic combination therapy is desired (Hammad and Shimamoto, 2010; Varankovich et al., 2015). In the study of Hammad and Shimamoto (2010), investigators deliberately screened for antibiotic-resistant probiotic strains in 40 commercially available Japanese probiotic supplements, which are to be used with a possible probiotic-antibiotic combination therapy. However, results showed no antibiotic resistance genes found in their isolates. As such, no isolated probiotic strain was reported feasible for a probiotic-antibiotic combination therapy.

Due to horizontal gene transfer, concerns are still raised particularly in lactic acid bacteria strains that carry mobile genetic elements such as plasmids (Varankovich et al., 2015). In the study of Mater et al. (2007), *in vivo* transfer of the vancomycin resistance gene, a plasmid encoded gene, was successfully demonstrated between lactic acid bacteria *Enterococcus faecium* strains and *Lactobacillus acidophilus* probiotic strains during digestive transit in mice. The results highlighted the risk of probiotics being a conduit for the spread of antibiotic resistance (Mater et al., 2007).

In the study of Chang et al. (2009), it was found that commercially available food and drugs with probiotic additives contained lactic acid bacteria strains positive for antibiotic resistance genes. Although the incidence of the antibiotic resistance genes was relatively low among the sample population, the antibiotic resistance genes found in the probiotic strains were located in mobile genetic elements such as plasmids and transposons. Despite the GRAS status of the specified lactic acid bacteria strains, the study’s findings confirm the threat of spreading antibiotic resistance genes through the use of probiotics. This is especially the case in countries without established guidelines and regulations in biosafety testing and rigid post-marketing surveillance. As such, a regulatory body, if not a united world-wide health authority, should always be in place to rigidly monitor probiotic use in every country (Chang et al., 2009).

### Bifidobacteria and Their Safety Profile

Another group of bacteria commonly used as probiotics belong to the genus *Bifidobacterium* (Gueimonde et al., 2013; Varankovich et al., 2015). *Bifidobacterium* is a known major constituent of the gut microbiota of both animals and humans. Bifidobacteria have the capacity to metabolize non-digestable host dietary carbohydrates such as plant-derived dietary fiber.

As a probiotic, strains of *Bifidobacterium* are known to inhibit adherence of enterotoxigenic *E. coli*, enteropathogenic *E. coli*, and *C. difficile* to intestinal epithelial cells (Varankovich et al., 2015). In combination with *Lactobacillus, Bifidobacterium* was shown to alleviate side effects of *H. pylori* eradication therapy as some *Bifidobacterium* strains also suppress *H. pylori*-induced genes in human epithelial cells (Varankovich et al., 2015). In addition, *Bifidobacterium* strains are also known to alleviate infectious diarrhea as well as inflammatory bowel disease (Varankovich et al., 2015). Like *Lactobacillus*, infectious diseases due to *Bifidobacterium* are extremely rare (Gueimonde et al., 2013).

*Bifidobacterium* strains are known to have intrinsic resistance against ciprofloxacin, nalidixic acid, mupirocin, streptomycin, and aminoglycosides (Wei et al., 2012; Gueimonde et al., 2013; Varankovich et al., 2015). However, resistance genes for lincosamides, macrolides, streptogramin B, and tetracycline are reported to be located in transposons (Gueimonde et al., 2013). In the study of Xiao et al. (2010), investigators tested the antibiotic susceptibility of *Bifidobacterium* strains distributed in the Japanese market. A total of 23 *Bifidobacterium* strains were isolated and tested for 15 antibiotics. Results confirmed the intrinsic resistance of *Bifidobacterium* against aminoglycosides. However, *Bifidobacterium animalis* subsp. *lactis* was found to be resistant to tetracycline and was in fact harboring a tetracycline resistance gene in its chromosome. The study concluded that although there is no risk factor for safety found in *Bifidobacterium* strains distributed in the Japanese market, the presence of the tetracycline resistance gene stresses the need for future evaluation (Xiao et al., 2010).

### Miscellaneous Concerns

The problem with the absence of a unified world-wide health authority that assumes responsibility for regulating probiotic use is exemplified by the case mentioned in the paper of Sanders et al. (2010). In countries with no established regulating body for probiotic use, the result is even worse as exemplified by the study of Chang et al. (2009). Keeping in mind that the likelihood of the spread of antibiotic resistance genes through human international travel was clearly indicated in the study of von Wintersdorff et al. (2014), we believe that a united global effort to screen probiotics that are marketed for human consumption is imperative.

In addition, given the established ecological nature of the emergence of antibiotic resistance problem, it is deemed logical to deduce that regulating probiotic use should also involve an ecological approach. It should be noted that studies involving the regulation of probiotic use in animals are lacking when compared to those of human applications. We should be reminded that numerous studies have already shown the connection between antibiotic resistance genes in animals and those in humans (Devirgiliis et al., 2011; Schjørring and Krogfelt, 2011; Forslund et al., 2013; Verraes et al., 2013; Allen and Stanton, 2014; Hu et al., 2014; Woolhouse et al., 2015). Without addressing regulations in animal probiotic applications, efforts in regulating human probiotic use might be considered inadequate in the end.

## Conclusion

**FIGURE 1:**
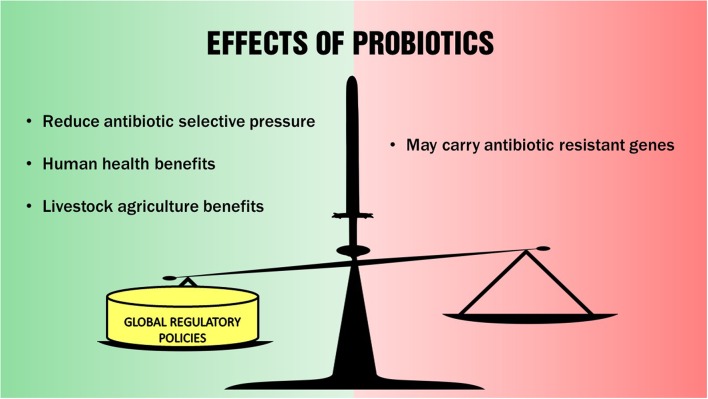
**Skewing the double-edged effects of probiotics toward positive outcomes**.

The emergence of antibiotic resistant pathogens through the spread of antibiotic resistance genes is an ecological problem that is exacerbated by the widespread indiscriminate use of antibiotics in livestock agriculture, and in veterinary and human medicine. The use of probiotics in lieu of antibiotics to control some diseases in animals and humans may reduce the antibiotic selective pressures on microorganisms in our natural environments and contribute in reducing the problem of the rapid emergence of antibiotic resistant pathogens. However, probiotic bacterial strains used in both animal and human applications also have risks in becoming conduits themselves in spreading antibiotic resistance genes. We conclude that the use of probiotics to address the global problem of emerging antibiotic resistant microorganisms is a “double-edged” sword – with both beneficial effects and associated risks as depicted in **Figure 1**. Therefore, although probiotics are currently generally regarded as safe, we think that it is imperative to implement proper regulation on their use in both livestock and human applications globally to effectively mitigate their potential contribution in the spread of antibiotic resistance genes in our natural environments.

## Author Contributions

II and JI conceptualized and wrote the manuscript.

## Conflict of Interest Statement

The authors declare that the research was conducted in the absence of any commercial or financial relationships that could be construed as a potential conflict of interest.
